# Probing Immobilization Mechanism of alpha-chymotrypsin onto Carbon Nanotube in Organic Media by Molecular Dynamics Simulation

**DOI:** 10.1038/srep09297

**Published:** 2015-03-19

**Authors:** Liyun Zhang, Xiuchan Xiao, Yuan Yuan, Yanzhi Guo, Menglong Li, Xuemei Pu

**Affiliations:** 1 Faculty of Chemistry, Sichuan University, Chengdu 610064, People's Republic of China; 2 College of Management, Southwest University for Nationalities, Chengdu 610041, People's Republic of China

## Abstract

The enzyme immobilization has been adopted to enhance the activity and stability of enzymes in non-aqueous enzymatic catalysis. However, the activation and stabilization mechanism has been poorly understood on experiments. Thus, we used molecular dynamics simulation to study the adsorption of α-chymotrypsin (α-ChT) on carbon nanotube (CNT) in aqueous solution and heptane media. The results indicate that α-ChT has stronger affinity with CNT in aqueous solution than in heptane media, as confirmed by more adsorption atoms, larger contact area and higher binding free energies. Although the immobilization causes significant structure deviations from the crystal one, no significant changes in secondary structure of the enzyme upon adsorption are observed in the two media. Different from aqueous solution, the stabilization effects on some local regions far from the surface of CNT were observed in heptane media, in particular for S1 pocket, which should contribute to the preservation of specificity reported by experiments. Also, CNT displays to some extent stabilization role in retaining the catalytic H-bond network of the active site in heptane media, which should be associated with the enhanced activity of enzymes. The observations from the work can provide valuable information for improving the catalytic properties of enzymes in non-aqueous media.

Over the past two decades, non-aqueous enzymatic catalysis has attracted considerable interests from experiments and theories since it can provide numerous synthetic and processing advantages compared to aqueous environment, for example, higher selectivity, thermo-stability, lower side reactions^1^,^2^,^3^. Thus, it could allow the syntheses of some compounds that are difficult to obtain in aqueous solution. Nevertheless, the enzyme in non-aqueous solution exhibits very low activity and lacks of long-term operational stability, which significantly limited its applications in industry. To tackle these problems, considerable experimental efforts have been devoted to activate enzymes in non-aqueous media, for example, salt activation, chemical modification and enzyme immobilization^4^. As known, the immobilization has been prevalently used in the classical enzymatic catalysis in aqueous media to improve the catalytic properties of enzymes^5^,^6^,^7^,^8^. Also, the method was adopted in the enzymatic catalysis in non-aqueous media, and the enhanced enzyme activity, stability and recyclability^4^,^9^,^10^,^11^,^12^ were reported. For example, glass-adsorbed chymotrypsin exhibited much higher activity than the suspended enzyme (by 1–2 orders of magnitude) in some polar and nonpolar organic media^10^. Subtilisin Carlsberg and α-chymotrypsin adsorbed onto silica support led to a significant increase in their activity in acetonitrile media with respect to the native enzyme preparation^11^. Lipases immobilized on poly(acrylonitrile-co-maleic acid) ultrafiltration hollow fiber membrane were observed to have higher hydrolytic activity and the operational stability than its free ones in heptane media^12^.

For the immobilization materials, solid supports^13^ were most widely used, which can be kinds of polymers, silica sol gels, hydrogels or nanoparticles. Compared to many flat supports, carbon nanotubes (CNTs) possess many advantages in improving the efficiency of biocatalysts owing to its unique electrical, mechanical and surface properties for the enzyme loading. Thus, CNT has been served as an excellent supporting material^14^,^15^,^16^,^17^ in aqueous and non-aqueous media. In addition, enzyme-CNT complexes not only represent a new generation of active and stable catalytic materials, but also have shown great potential applications in field of biosensors^18^,^19^, biomedical devices^17^ and other hybrid materials^20^.

Although the enzyme immobilization has been an effective strategy to improve the catalytic properties of enzymes in aqueous phase, the mechanism of immobilization at the microscopic level has been not satisfactorily clarified in experiments owing to the complexity of the system and the molecular nature of the issue. Molecular dynamics (MD) simulations can reveal the microscopic nature of intermolecular interactions. Thus, the method has been successfully used to study the immobilization of enzymes in aqueous solution. Li^21^ used MD simulations to investigate the interactions between chymotrypsin (ChT) and graphene/graphene oxide (GO) and found that GO exhibit good capability to inhibit ChT. Szleifer^22^ investigated the adsorption process of lysozyme onto a polyethylene (PE) surface in aqueous environment via a large-scale atomistic molecular dynamics simulation. Cai^23^ performed the MD study on the immobilization of papain. The adsorption process of human serum albumin onto an ion-exchange chromatographic medium was observed to be faster, stronger than bovine hemoglobin through MD simulations^24^. MD simulations were also used to study interactions of single-walled CNTs with lysozyme^25^, human serum albumin^26^, protein domains^27^,^28^, kinds of peptides^29^,^30^ and cosolvents^31^ in aqueous solution. Despite of the growing number of computational studies dealing with the solid supports and the proteins in aqueous phase, the studies have been seriously lacked in non-aqueous media so that the understanding of immobilization mechanism in the media has been lagged, which significantly disfavor the effective application of the immobilization method in practice. Based on these considerations above, we, herein, performed all-atom MD simulations to study the interaction between α-chymotrypsin (α-ChT) and CNT in non-polar heptane media and aqueous solution. As known, the chymotrypsin is a member of the serine protease family and one of the most widely used enzymes in peptide bond synthesis^32^. Also, it has been used for peptide and amino acid ester syntheses in different organic solvents at low water content^33^,^34^. More importantly, the immobilization method was demonstrated by some experiments to be effective in enhancing the activity and stability of α-ChT in non-aqueous media^10^,^17^. Heptane was chosen in this work since it is one of the most common nonpolar organic media for non-aqueous enzymatic catalysis^35^,^36^. In previous work^37^,^38^, we already used MD simulation to study the effects of the organic solvent and water content on the structure and function of the chymotrypsin. In the work, we further revealed the molecular mechanism concerning the effect of the immobilization of CNT on its structure and stability in the non-polar heptane media, in order to provide valuable information for improving its catalytic properties and advancing its application in non-aqueous media.

## Results and Discussion

In the work, two different solvent environments were considered, viz., heptane media (labeled as HEP) and water solution (labeled as WAT). Four different systems were constructed. One is α-ChT immobilized to one single walled carbon nanotube in heptane media, labelled as cnt-hep. The second is α-ChT immobilized to the single walled CNT in aqueous solution, labeled as cnt-wat. As a reference, we also set up the free enzyme systems in aqueous solution (labeled as free-wat) and heptane media (labeled as free-hep). MD simulation times are 200 ns for every system. In order to be in line with the experimental procedures^17^,^36^ in which the enzymes were first immobilized on carbon nanotubes in aqueous solution and then was placed into the organic solvents, we extracted the final snapshot of the complex of the enzyme with CNT from the 100 ns trajectories in aqueous solution as the starting structure to simulate the immobilized enzyme in heptane media. Three parallel 200-ns simulations with different starting velocities were performed for each system in order to enhance sampling space and assess the reproducibility of the results. Similar results were always observed and there are no significantly different binding modes between these parallel simulations (see Supplementary Fig. S1). Thereby, in the following, we only selected one representative trajectory from every system to discuss in detail.

### Adsorption process and overall structure changes

**Figure 1:**
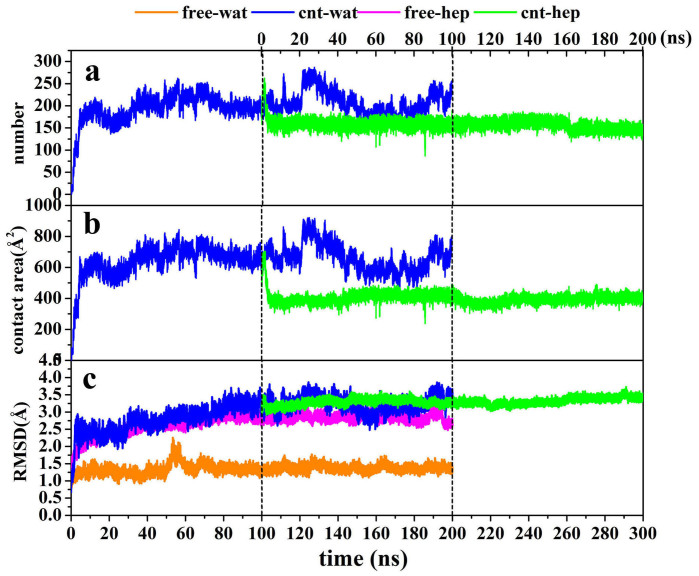
(a) The changes in the RMSD values of backbone atoms of α-ChT, (b) the number of adsorbed atoms and (c) the contact area between the CNT and the enzyme with respect to 200 ns simulation time in aqueous and heptane media for the free and immobilized enzymes. The RMSD is for deviation from the crystal structure. The initial structure of the enzyme-CNT complex in cnt-hep system is derived from the last snapshot of the 100 ns trajectories in cnt-wat system.

In order to quantify the extent of the protein adsorption on CNT in the two different solvents, we calculated the number of adsorbed non-hydrogen atoms within 6 Å distance from the surface (Fig. 1a) since the distance is generally served as a criterion of hydrophobic interaction^26^. In addition, we also calculated the contact area between the CNT surface and α-ChT (Fig. 1b) in terms of Eq. (1), serving as another indicator to measure the adsorption extent.
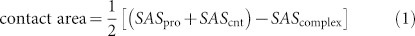


*SAS*_pro_, *SAS*_cnt_ and *SAS*_complex_ in Eq. (1) denote the solvent accessible surface area of the isolated protein, CNT and the CNT-protein complex, respectively.

To gain insight into the impact of the adsorption extent on the overall structure of the enzyme, we also calculated root-mean-square deviation (RMSD) of the enzyme from its crystal structure. The three parameters calculated were plotted as a function of time in Fig. 1c. It is not surprised that there are similar change trends for the three parameters. In the initial 5 ns for the enzyme in aqueous solution, the number of atoms adsorbed and the contact area sharply increase up to be ~200 and ~600 Å^2^, respectively. The result indicates a quick and strong adsorption of the enzyme to the surface of CNT in aqueous solution, which are evidenced by the distinct increases in the RMSD values within the initial 5 ns. In the following 25 ns, the three types of indicators do not continuously present a significant increase but experience small fluctuations, indicating a relatively stable stage in the adsorption process. After that, they also begin to slightly increase until 40 ns and then almost approach to an equilibrium with small fluctuations in the remaining 160 ns simulation time for the aqueous media.

As mentioned above, for the adsorption in heptane media, we selected the last snapshot of the immobilized α-ChT from the 100 ns simulation trajectories in aqueous solution and placed it into the heptane media as an initial conformation (see green lines in Fig. 1). Figure 1 clearly shows that the number of atoms adsorbed and the contact area for the immobilized α-ChT goes downhill significantly from ~220 to ~150 and from ~600 Å^2^ to ~400 Å^2^ in the first 4 ns simulation in the heptane media, respectively, accompanying with slight decreases in the RMSD value. The observation implies an occurrence of desorption. And then, the number of the adsorbed atoms and the contact area soon tend to an equilibrium with tiny fluctuations in the remaining simulation time while the RMSD value of the immobilized α-ChT still exhibits a slow and slight increase, which should be induced by the non-polar heptane solvent. The average values over the last 20 ns trajectory in heptane media are calculated to be 148 for the number of the adsorbed atoms and 405 Å^2^ for the contact area, much smaller than those in aqueous solution (211 and 615 Å^2^). The observation clearly reflects the quick desorption behavior of the enzyme upon the change of solvent environment from the polar aqueous media to the non-polar heptane one. The average RMSD values of the immobilized α-ChT over the last 20 ns are 3.35 Å in aqueous solution and 3.42 Å in heptane solvent, which are close to each other. In addition, Fig. 1c also displays the RMSD change for the free α-ChT in the aqueous solution and the heptane media. An inspection of Fig. 1c shows that the RMSD value of free α-ChT in either aqueous solution or non-polar heptane one are smaller than those of immobilized α-ChT in corresponding solvents. The average RMSD values of the free α-ChT over the last 20 ns trajectories are 1.37 Å in aqueous solution and 2.81 Å in heptane media. The differences in the RMSD value between the free enzyme and the adsorbed enzyme over the last 20 ns trajectories are 1.98 Å in aqueous solution and 0.61 Å in heptane media. The difference indicates that the impact of CNT adsorption on the structure of α-ChT is more significant in aqueous solution than heptane media.

**Figure 2:**
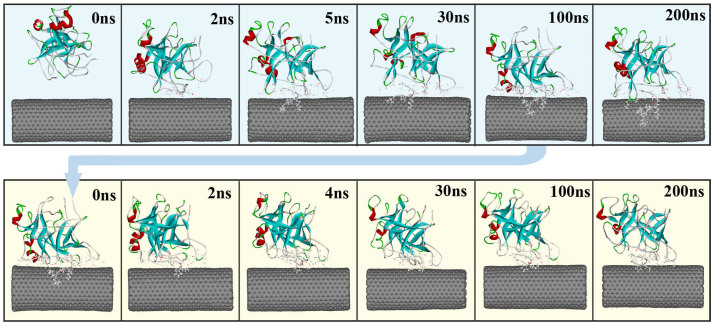
The representative snapshots selected from the 200 ns simulation trajectories on basis of the RMSD variation of the α-ChT in aqueous (Top) and heptane (Bottom) media for the immobilized enzymes. α-ChT is displayed in ribbon style and colored by secondary structure type. The CNT is displayed in CPK style. The residues within 6 Å of the CNT surface are displayed in line style. The initial structure of the enzyme-CNT complex in cnt-hep system is derived from the last snapshot of the 100 ns trajectories in cnt-wat system.

**Figure 3:**
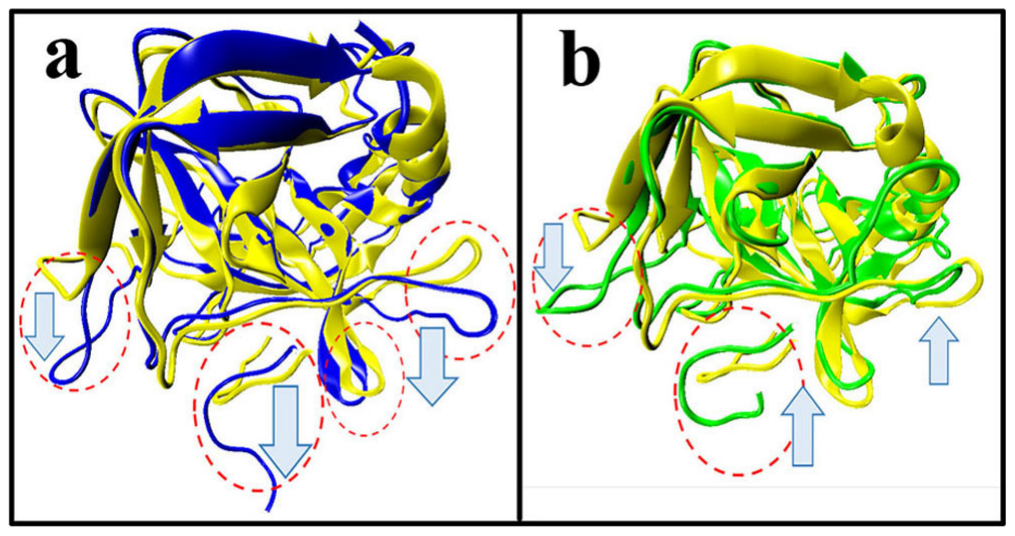
Superposition of structures with the lowest RMSD value from the corresponding average one of immobilized α-ChT in (a) aqueous and (b) heptane media over the last 20 ns trajectories with the crystal structure. The arrows indicate the tendency of mobile segments. Regions with significant changes are circled in red. Color code: yellow, crystal structure; blue, the immobilized α-ChT in aqueous solution (cnt-wat); green, the immobilized α-ChT in heptane media (cnt-hep).

In order to visually view the variation of protein conformation upon adsorption, we selected some representative snapshots on the basis of the RMSD variation to display the adsorption process in the two media. Figure 2 clearly shows that the rapid adsorption process indeed occurs in the initial 5 ns in aqueous environment. In the following 25 ns, no significant changes in the distance between the enzyme and CNT are observed, instead of to some extent spreading of α-ChT along the CNT surface. And then, the enzyme further undergoes a conformational rearrangement to maximize its interactions with the tube surface until 200 ns in aqueous solution in order to stabilize the enzyme-CNT binding. When the immobilized α-ChT in aqueous solution (viz., the last snapshot of 100 ns trajectories in aqueous solution) was placed into heptane media, to some extent desorption could be observed in the initial 4 ns in heptane media, as reflected by Fig. 2. After that, no significant changes are observed for the overall structure of the enzyme in the subsequent adsorption process, where the enzyme further optimized its interaction with CNT in heptane media. Thus, we used the snapshots from the 30^th^ ns, 100^th^ ns and 200^th^ ns trajectories in heptane media to reflect the remaining adsorption process. In addition, Fig. 3 displays a superposition of the crystal structure and the representative snapshots of the protein with the lowest RMSD value from the average structure in the two solvents. It is distinct that the structures of coils and turns of α-ChT near CNT were significantly pulled down compared to the initial crystal structure, reflecting the strong adsorption interaction in aqueous solution. Whereas for heptane media, it is observed from Fig. 3 that some mobile segments were escaped away from the CNT surface, clearly exhibiting to some extent desorption. These observations from Fig. 2–3 are consistent with those from the number of adsorbed atoms, the contact area and the RMSD values.

**Figure 4:**
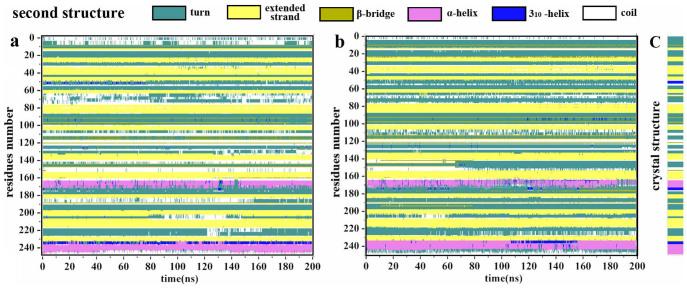
Evolution in time of the secondary structure of the per-residues of immobilized enzyme in (a) aqueous solution and (b) heptane media. (c) The crystal secondary structure as a reference.

To gain insight into the effect of the adsorption on the second structures of α-ChT in the two different media, the STRIDE (called as the structural identification method)^39^ program was used to calculate the change trend of the second structures along the adsorption process, as shown in Fig. 4. A comparison of the immobilized α-ChTs in the two solvents indicates that the adsorption plays no significant part in influencing the primary secondary structures like helix and sheet, and α-ChT almost maintains its native conformation despite of the adsorption of CNT in either aqueous solution or the non-polar solvent. Experimental and theoretical investigations^25^,^40^ on lysozyme, horseradish peroxidase and subtilisin Carlsberg adsorbed onto nanotubes in aqueous solvent also found that the secondary structure and the majority of the tertiary structure can be preserved and only coils and turns structures are affected, in line with our results. These observations suggest that the rearrangements of the residues upon adsorption are limited either in aqueous solution or in organic media, and the protein strives to preserve its secondary structure, which should facilitate the enzyme to maintain its catalytic activity.

### Binding energy analysis and key residues contributed to the adsorption

To study the thermodynamics of the physical adsorption of α-ChT on CNT in the two media, we performed molecular mechanics generalized Born/surface area (MM/GBSA)^41^ analysis to estimate the binding energy. The binding free energy *ΔG*_binding _was calculated using Eq. (2)
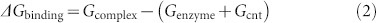


Where *G*_complex_, *G*_enzyme_ and *G*_cnt_ are the free energies of the complex, the enzyme, and CNT, respectively. The free energies are estimated as a sum of four terms (see Eq. 3)
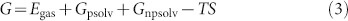


Where *E*_gas_ is the gas-phase energy and expressed as the sum of the internal energy (*E*_int_), the electrostatic (*E*_ele_) and van der Waals (*E*_vdw_) interaction energies. *G*_psolv_ and *G*_npsolv_ denote the polar and nonpolar contribution to the solvation free energy, respectively. *T* denotes the absolute temperature and *S* is the molecule entropy. Similar to many computational investigations^42^,^43^,^44^, the free energy calculations in the work do not consider the change in solute entropy since we more concerned with the relative change of the binding affinity.

The GB model (IGB = 2) and the LCPO method (see Eq. 4) was used to calculate *G*_psolv_ and *G*_npsolv_ terms, respectively. SASA represents solvent-accessible surface area.
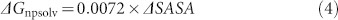


The value of 1 was used for the interior dielectric constant of the enzyme while values of 80 and 1.92 were used for the exterior solvent dielectric constant for water and heptane, respectively. One thousand snapshots without the water and organic molecules as well as chloride ions were extracted from the MD trajectories in the last 20 ns at 20 ps intervals for the energy analysis.

Table 1 lists the calculated energy and its components in the two solvents. Negative Δ*G*_binding_ values in Table 1 represent that the adsorption of the enzyme on CNT is spontaneous in the two media. Data in Table 1 shows that the binding energy in heptane media (−47.53 ± 3.31 kcal mol^−1^) is significantly smaller than that in aqueous solution (−83.65 ± 4.37 kcal mol^−1^), indicating much weaker adsorption in the non-polar organic media. The result is also consistent with less atoms adsorbed and smaller contact area in heptane media with respect to aqueous solution, as revealed above. Further analysis on the binding components of the energy reveals that there is no contribution from non-bonded electrostatic interaction to the adsorption (*ΔE*_ele_ = 0.0 kcal mol^−1^). Main driving forces to the adsorption are van der Waals (*ΔE*_vdw_) interactions in the polar and non-polar media, providing a sound support for the hydrophobic interaction between proteins and CNTs. The absolute value of van der walls interaction is much larger in aqueous solution (−122.07 ± 5.66 kcal mol^−1^) than that (−67.54 ± 2.55 kcal mol^−1^) in heptane media. The disfavor contribution from the polar solvation free energy to the adsorption is larger in aqueous solution (48.43 ± 2.53 kcal mol^−1^) than heptane media (25.96 ± 2.51 kcal mol^−1^) and partly offsets the favorable contribution from van der walls interaction. But, the total binding energy in aqueous solution is significantly higher than that in heptane media.

**Figure 5:**
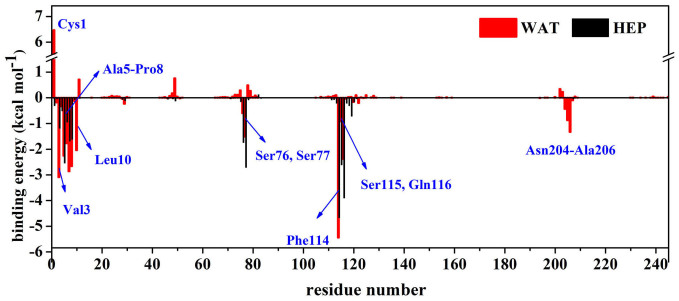
Per-residue binding energy for the CNT binding in aqueous (WAT) and heptane (HEP) media. Some residues, which have significantly different contribution to the binding between the two media, are labelled.

**Figure 6:**
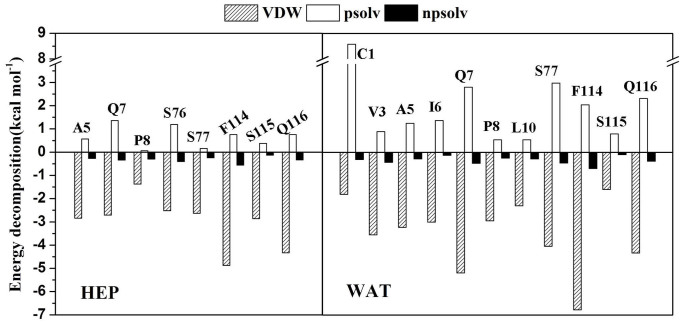
Energy decomposition into contributions from van der Waals energy (VDW), the polar solvation energy (psolv) and the nonpolar term of solvation energy (npsolv) for the residues whose absolute value of binding energy was greater than 1.5 kcal mol^−1^. WAT denotes aqueous solution and HEP denotes heptane media.

In order to further identify important residues contributed to the adsorption, we also decomposed the binding energy on a per-residue basis to obtain the contribution from structural elements (see Fig. 5). Figure 6 further displays the energy decompositions of high-energy residues with the absolute values of binding energies greater than 1.5 kcal mol^−1^.

**Figure 7:**
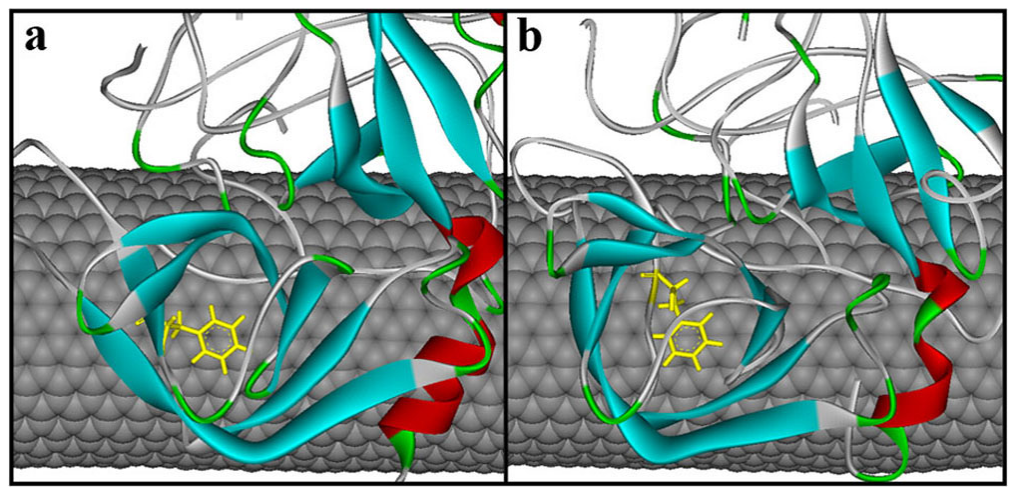
The π-π interaction between the CNT surface and the residue Phe114 in (a) aqueous solution and (b) heptane media.

As revealed by Fig. 6, the residues Val3, Ala5, Ile6, Gln7, Pro8, Leu10, Ser77, Phe114, Ser115 and Gln116 are observed to significantly contribute to the binding energy mainly through van der Waals interactions in aqueous solution. It is not unexpected since most of these residues, such as Val3, Ala5, Ile6, Pro8, Leu10 and Phe114, are hydrophobic residues. The most significant contribution to the adsorption comes from the residue Phe114 (−5.44 kcal mol^−1^). Phe114 is located in the C-terminal and has high mobility. It is observed from Fig. 7 that Phe114 residue parallels to the CNT surface, thus forming π-π stacking interaction with CNT. The observation is consistent with findings from some previous works^28^,^45^ that the π-π stacking interaction plays an important role in the interaction between proteins and these nanomaterials.

However, when put the immobilized enzyme from aqueous solution into heptane media, significant changes in the number of favorable residues and their contribution extent occur. In heptane media, the residues Val3, Ile6, Gln7, Pro8 and Phe114, which are observed to favor the binding in aqueous solution, still retain their favorable contribution (see Fig. 5–6). But the contribution extent are decreased due to the change in the solvent environment, as evidenced by the differences in their energy values between aqueous solution and heptane media. Meanwhile, the non-polar heptane media increases the favorable contribution extent from the residue Ala5, Ser76, Ser77, Ser115 and Gln116 with respect to aqueous environment. But, the favorable contribution from the residues Leu10, Asn204-Ala206 are disappeared in heptane media.

**Figure 8:**
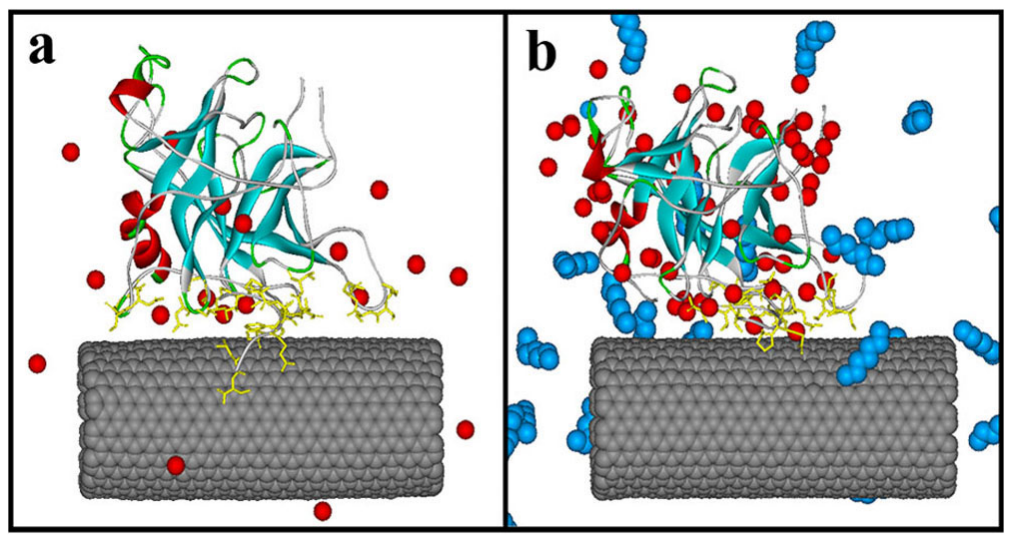
The distribution of solvent molecules around the immobilized enzyme in (a) aqueous solution and (b) heptane media. Only crystal water molecules and the heptane molecules close to the surfaces of the enzyme and CNT are showed as CPK model in red and blue, respectively.

As revealed by Fig. 8, some heptane molecules, diffuse into the contact region between the enzyme and CNT. These heptane molecules could compete with residues to interact with CNT (see Fig. 8) and make some residues Cys1, Gly2, Leu10, Ala126-Asp128, Asn204-Ala206, which are very close to the CNT surface in aqueous solution, escape from the CNT surface, thus leading to less adsorbed atoms and contact areas in heptane media. It is consistent with the observations above that the residues close to the CNT in aqueous solution are pulled up and become far from the CNT. Some studies^46^,^47^,^48^ on adsorption of organic compounds on CNTs in aqueous phases also found that the non-polar solvent molecules are more likely to attach with CNTs than water molecules due to the driving force of the hydrophobic interaction, in line with our observations. It is the competition from the non-polar heptane molecules that significantly weaken the binding energy between α-ChT and CNT in heptane media relative to aqueous solution.

### Changes in local regions of α-ChT

#### Structure changes in three polypeptide chains

**Figure 9:**
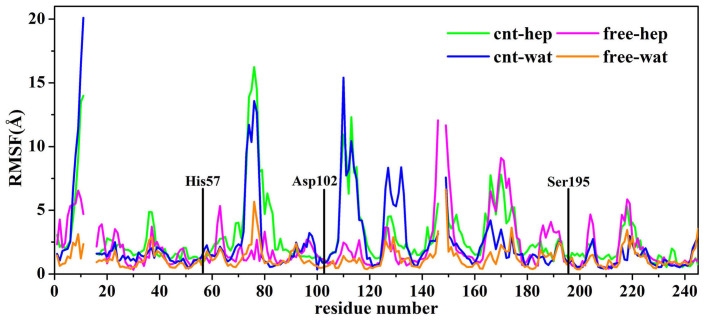
A comparison of average RMSD values of per-residue of the enzyme between aqueous solution and heptane media over the last 20 ns trajectories in the free and immobilized states. The RMSD is for deviation from the crystal structure. The three catalytic residues are marked in black lines.

α-ChT is composed of 245 residues, which contains three separated polypeptide chains (chain A: residues1-11, chain B: residues16-146, chain C: residues149-245) linked by five disulfide bridges. We calculated the root mean square fluctuation (RMSF) value of each residue and displayed the result in Fig. 9. As expected, residues located at the terminal of side chain exhibit high RMSF values in the free and immobilized systems, due to their high flexibility. In the two immobilized systems, the residues located at the chain A and chain B, close to the CNT surface, also display large RMSFs owing to the adsorption from CNT. In addition, high RMSF values are observed for the residues Cys1-Ser11, Ser71-Glu74, Ser109-Thr113, Asa122-Asp124, which are close to CNT surface. The RMSF values of the chain C of the free α-ChT are larger than the other two chains in heptane media since majority residues of the chain C are located at the surface of enzyme so that they can easily contact with the organic solvent. However, these residues of the immobilized α-ChT exhibit lower RMSF values in heptane media, implying that carbon nanotubes could to some extent improve stability of the enzyme in the organic media. In contrast, the stabilization impact of CNT on the chain C is not observed in aqueous solution.

#### The catalytic triad

**Figure 10:**
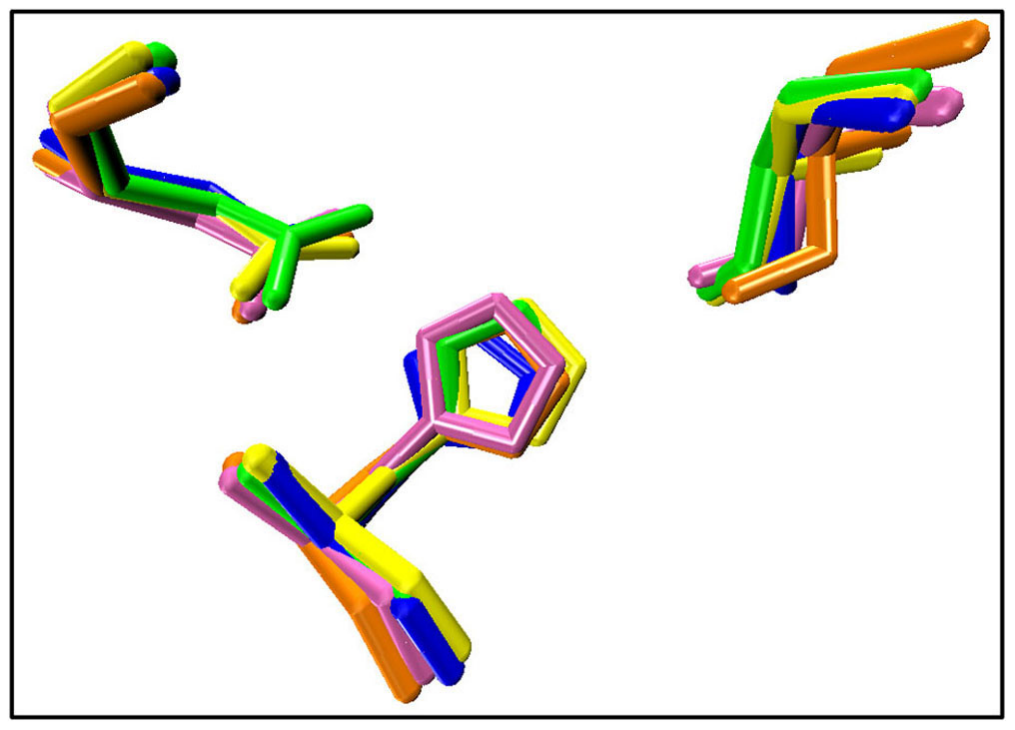
A structure superimposition of the three catalytic residues of the enzyme between the crystal structure and the structures with the lowest RMSD value from the corresponding average one derived from the MD simulation trajectories in four systems. Hydrogen atoms are not shown. Color code: yellow, crystal structure; blue, cnt-wat; green, cnt-hep; orange, free-wat; pink, free-hep.

The catalytic triad of α-ChT is composed of three residues (His57, Asp102, and Ser195). We could expect that it will be difficult for an enzyme to maintain optimal activity if the conformation of active site is distorted. As shown in Fig. 9, the three catalytic residues exhibit much lower RMSF values than most residues of the enzyme in all systems. A superimposition of the three catalytic residues between the four systems and the initial crystal structure further confirm the small changes in the conformation of the catalytic triad, as reflected by Fig. 10. The observation suggests that the catalytic residues could keep relative rigidity, insensitive to the solvent environment and the adsorption of CNT, which should be associated with the fact that the enzyme could retain its catalytic activity in organic media and immobilization environments^10^,^11^,^12^.

As revealed, H-bond network between the three active residues plays an important role in the catalytic reaction of the serine proteases^32^. Hence, we carried out a detailed analysis on the H-bonding (see Table 2). We considered one hydrogen bond to be formed if both the Donor **···** Acceptor distance is less than 3.5 Å and the angle between the hydrogen bond donor atom, hydrogen atom, and acceptor atom is more than 120°. If a hydrogen bond exists for >90% of the trajectory time, we will consider it as a stable one. As can be seen from Table 2, the H-bonding between the Asp102 and the His57 residues is very stable, insensitive to the adsorption of CNT and the solvent environment. In contrast, the solvent and CNT could significantly influence the H-bonding between His57 and Ser195 residues. There is a significant difference in the percentage occupation of the H-bond of NE2@His57**···**OG@Ser195 between the two solvents. The CNT adsorption in aqueous solution almost breaks the H-bond, implying an unfavorable effect on the catalytic activity of the enzyme. Indeed, some experiment findings reported that the catalytic activity of enzymes is decreased by the immobilized state in aqueous solution^4^,^32^. Surprisingly, CNT in heptane media significantly stabilize the H-bonding between Ser195 and His57 with life time of 99.7%, which should be partly associated with some experimental facts that the glass immobilized α-ChT^10^ and multiwalled carbon nanotubes immobilized lipase^36^ have higher bioactivity than that in free type in organic solvents. In addition, the hydrogen bond forms more frequently for the immobilized α-ChT than the free one in heptane media. As reflected by Supplementary Fig. S2, one crystal water molecule was observed to penetrate into the active site and intermittently form hydrogen bonding with OG@Ser195 for the free enzyme in heptane media, in which the percentage occupation of the H-bond was calculated to be 43.8% during the last 20 ns trajectories. In contrast, there was no crystal water molecules penetrating into the active site of the immobilized α-ChT in heptane. It should be the perturbation of the water molecule that contributes to less stable H-bonding between Ser195 and His57 residues in the free enzyme than the immobilized one in heptane media.

#### S1 Specificity pocket

**Figure 11:**
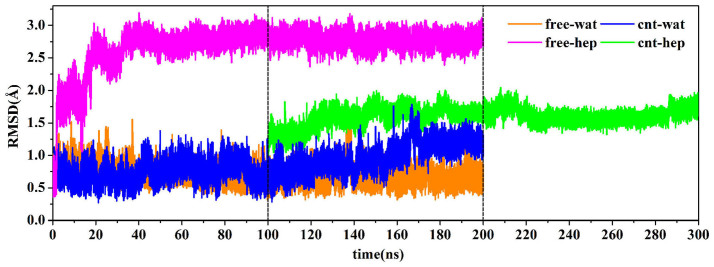
The changes in the RMSD values of backbone atoms of S1 pockets with respect to the 200 ns simulation times in four different systems, The RMSD is for deviation from the crystal structure. The initial structure of the enzyme-CNT complex in cnt-hep system is derived from the last snapshot of the 100 ns trajectories in cnt-wat system.

The S1 pocket of the enzyme, which is adjacent to the surface and bordered with the side chains of residues 189–195, 213–220 and 226–228, was revealed to be associated with the specificity of serine proteases^32^. Thus, we focused on the structure changes in the S1 specificity pocket. The RMSD variation of S1 pocket backbone atoms along time are depicted in Fig. 11. As reflected in Fig. 11, the highest RMSD value of S1 pocket is observed for the free enzyme system in heptane media compared to the other three systems, implying that the specificity of the free enzyme in heptane media should be significantly changed. But, when α-ChT is physically immobilized on CNT in the non-polar organic solvent, the RMSD value of S1 pocket is significantly decreased relative to the free type in the same media, although it is slightly larger than those in aqueous solution. The observation exhibits the stabilization effect of CNT on the S1 pocket of α-ChT in non-polar heptane media. A comparison between the free α-ChT and the immobilized one in aqueous solution suggests that the CNT adsorption in aqueous solution plays no significant role in the S1 pocket conformation although it still slightly increases the RMSD value, as shown in Fig. 11.

**Figure 12:**
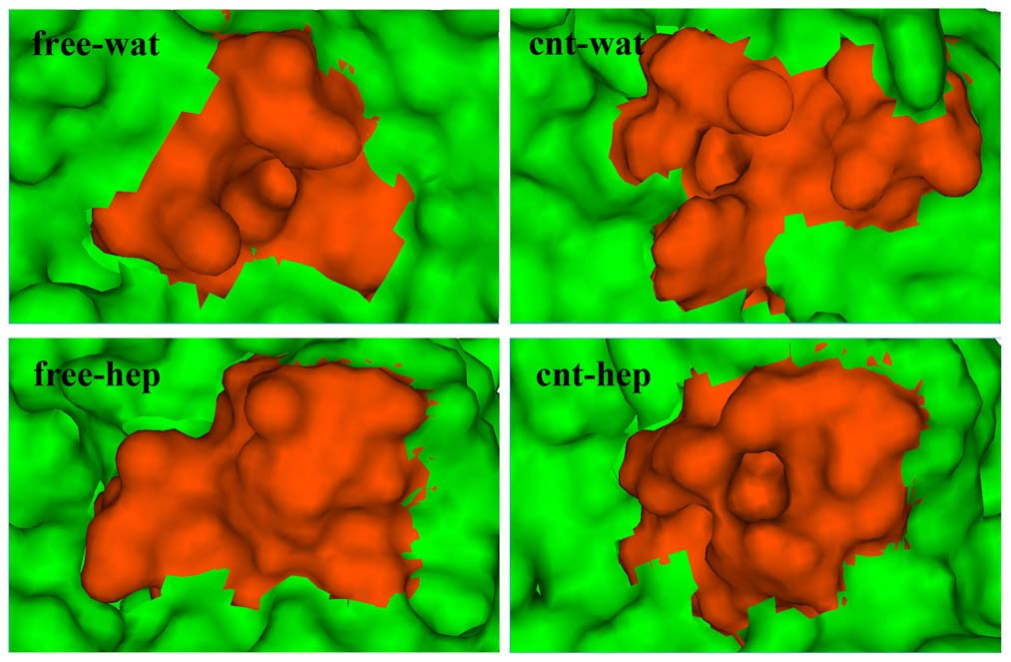
The structures with the lowest RMSD value from the corresponding average one of S1 pockets over the last 20 ns trajectories in four different systems. Red and green denote the residues of the S1 pocket and the other residues around the S1 pocket, respectively.

**Figure 13:**
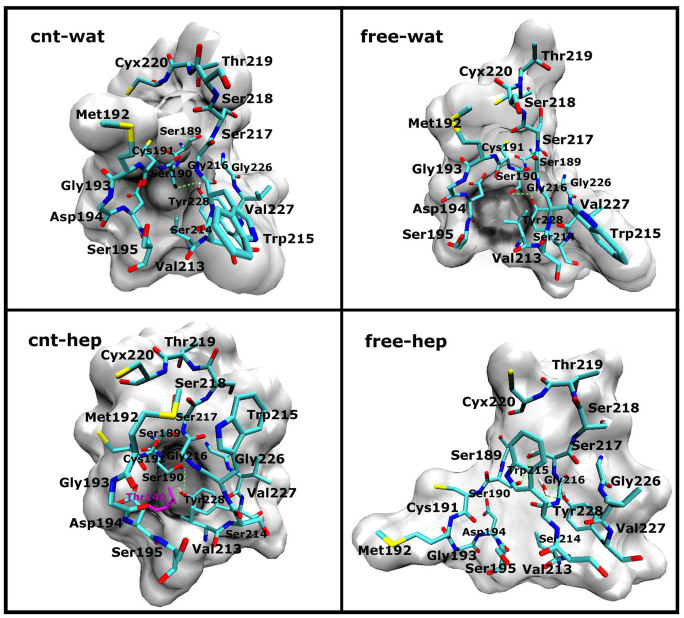
Key H-bonding in the S1 pocket for the four different systems, derived from the frame with the lowest RMSD value from the corresponding average structure over the last 20 ns trajectories. The S1 pocket residues are shown by both surface and stick models. The key hydrogen bonds are shown by green dotted line.

In order to visually observe the effects of the organic solvent and the adsorption on the S1 specificity pocket, we display the structures of S1 pockets in the four different environments in Fig. 12, which is derived from the frame with the lowest RMSD value from the corresponding average structure over the last 20 ns simulation trajectories. As observed from Fig. 12, there are no large differences in the S1 pocket structures in aqueous solution either with or without CNT, almost preserving the initial shape of the pocket with an obvious cavity. The observation implies that the adsorption of CNT would not significantly influence the specificity of α-ChT in aqueous solution. However, the S1 pocket of the free enzyme in heptane solvent is almost fully closed and distinctly different from those in aqueous solution, implying a change in the specificity of the enzyme, consistent with the experiment finding^38^,^49^. In contrast, the shape of the S1 pocket for the immobilized enzyme in heptane solvent still presents relative “open” state, similar to those in aqueous solution. To probe into the molecular mechanism on the differences in the S1 pocket conformation between the four different environments, we further calculated the H-bond interactions between the residues in the S1 pocket and H-bonding between the residues and the solvent molecules. The key hydrogen bonds are shown in Supplementary Table S1 and Fig. 13. The hydrogen bond of OG@Ser190**···**OH@Tyr228 links the two regions of S1 pocket (see Fig. 13), which consist of Ser189-Ser195 residues and Gly226-Tyr228 residues, respectively. While the H-bond was kept during more than half simulation time for the free and immobilized enzymes in aqueous solution, thus stabilizing the conformation of S1 pocket. When shifted from aqueous solution to the hydrophobic heptane solvent, the hydrophobic residues located at the inner of pocket were overturned to the outside for the free enzyme system. As reflected by Fig. 13, the residues Ser189-Gly193 were completely extended for the free enzyme in heptane media with respect to the systems in aqueous solution. Accordingly, the hydrogen bond of OG@Ser190**···**OH@Tyr228 was fully broken, as evidenced by Supplementary Table S1. Alternatively, Ser190 forms H-bond with Trp215 and Tyr228 forms H-bond with one crystal water molecule. As a result, the significant changes in S1 pocket conformation occurred and lead to the nearly “close” state of S1 pocket for the free enzyme in heptane media. Figure 9 also confirms that the RMSF values of these residues above are much greater in the free-hep system than the other three systems, providing a support for the observation above. Whereas for the immobilized enzyme in heptane media, the H-bonding between OG@Ser190 and OH@Tyr228 was still partly kept during the last 20 ns simulation time, as evidenced by its percentage occupation of 43.8%, although it was to some extent weakened by the hydrophobic solvents derived from its lower percentage occupation than those in aqueous solution (see Supplementary Table S1). In addition, the residues Thr140 of the immobilized enzyme in heptane media, near to the Ser190 residue, also forms H-bonding with Tyr228 (the life time of 84.5%), which should play to some extent role in stabilizing the S1 conformation. Thus, the S1 pocket of the immobilized enzyme still keep relative “open” state in heptane media, different from the free enzyme in the same media. The observation further confirm that the CNT adsorption significantly stabilize the S1 pocket structure in the non-polar media, providing a molecular support for the experiment finding^8^,^50^ that the immobilization of enzyme in organic solvents could to some extent keep its initial specificity.

## Conclusion

In the work, we used all-atom molecular dynamics simulation to study the adsorption of α-ChT on CNT in aqueous solution and heptane media in order to shed light into the molecular mechanism concerning the effect of the immobilization on improving catalytic properties of enzyme in non-aqueous media. As a reference, the free α-ChT in the two types of media was also simulated in the work.

The results indicate that α-ChT can be strongly absorbed onto CNT in aqueous solution, but the adsorption would be weakened in the non-polar heptane solvent, as evidenced by less adsorption atoms and larger contact area as well as higher binding free energies in aqueous solution than those in heptane media. The van der Waals interaction is revealed to be main driving force to the adsorption. Some heptane molecules diffuse into between the enzyme and CNT, and compete with the α-ChT to interact with CNT, which result in to some extent desorption and the significant drop in binding strength.

Not surprisingly, there are larger changes in the overall structures of α-ChTs attached to CNT with respect to the free ones in both aqueous solution and heptane media, which mainly stem from the impact of the CNT adsorption. But, no significant changes in secondary structure of the enzyme upon adsorption are observed in aqueous solution and non-polar heptane solvent. Interestingly, the structures of some local regions of α-ChTs in the immobilized state (for example, the chain C and the S1 pocket) have smaller RMSD values in heptane media than those of the free enzyme system, displaying stabilization effects of CNT on the local regions of α-ChT in non-polar organic media. In contrast, the stabilization effect could not be observed in aqueous solution. In addition, the catalytic residues are insensitive to the organic environment and the adsorption of CNT. CNT also displays to some extent stabilization role in retaining the catalytic H-bond network of the active site in heptane media, different from aqueous solution. These observations provide useful information at the molecular level for understanding the activation mechanism regarding the effect of the immobilization on the activity, stability and the specificity of enzymes in non-polar solvent environment. We hope that the work can be helpful for improving the catalytic properties of enzyme in organic media.

## Computational methods

All MD simulations were carried out using the AMBER 12 package^51^. The ff99SB^52^ force field was used for alpha-chymotrypsin (α-ChT).

### Construction of models

The initial coordinate of native crystallized α-chymotrypsin was obtained from the diffraction data with 1.68 Å resolution (the PDB entry code is 4CHA^53^), which contains two independent α-chymotrypsin molecules in the crystallographic asymmetric unit. So, we removed one of the enzyme molecules and the corresponding water of crystallization. The final coordinate of α-chymotrypsin contains 239 residues and 50 water molecules. The single walled carbon nanotube with chirality (14, 14) was built using VMD package^54^, in which the C-C bond length is defined as 1.42 Å and pipe diameter size is about 1.9 nm. We choose one single walled CNT with length of about 5.0 nm to fit the size of chymotrypsin, which contains 1176 carbon atoms. Similar to some previous studies, the carbon atoms of CNT were modeled as uncharged lennard-jones particles^55^ using sp2 carbon parameters of the ff99SB force field.

Initially, the minimum distance between the α-chymotrypsin and the CNT surface is more than 12 Å. The carbon nanotubes can produce a strong adsorption as they approach the active site, which can influence the bioactivity of enzymes^27^. Thus, we placed the hydrophobic surface of the enzyme facing the CNT while the active site of the enzyme is opposite to it. Extra water and heptane molecules were added using the LEAP utility. All systems were then solvated in a rectangular water box with periodic boundary conditions, using TIP3P^56^ water model. The sizes of the water boxes are 75.71*80.73*67.30 (Å^3^) with 8899 water molecules for the cnt-wat system and 79.09*68.06*68.90 (Å^3^) with 8761 water molecules for the free-wat one. The initial coordinates of the heptane molecules were optimized of the heptane molecule was optimized at the HF/6-31G* level using Gaussian 09^57^. The partial charge was estimated using the RESP program of AMBER12.0. The GAFF force field was used for the heptane molecules. The number of heptane molecules we added to the free-hep system is 1189 and the size of the heptane box is 74.52*71.72*80.39 (Å^3^). For the enzymes in heptane media, we retained about 50 water molecules directly interacted with the protein and then added 1216 heptane molecules with the box size of 74.52*76.85*88.58 (Å^3^). Chloride ions were introduced to remain neutral of the whole system.

### Simulation details

We use the same optimization steps and parameters in MD simulation for the four systems. All initial configurations were optimized by a series of energy minimizations: first only keeping the solvent fixed, and then keeping the enzyme and CNT fixed, and finally setting free the whole system. At 5000-step optimizations, the steepest descent and conjugated gradient algorithms were used for the first 3000 steps and the remaining 2000 steps, respectively. After that, the systems were warmed up from 0 to 300 K within 120 ps using the Berendsen temperature coupling^58^. Then 5 ns dynamics simulations were carried out in the canonical (NVT) ensemble at 300 K. Finally, 195 ns subsequent MD simulations without any restrictions were carried out in the isothermal isobaric (NPT) ensemble. The integration step for MD simulations was set to 2 fs. The particle mesh Ewald (PME) method^59^ was applied to treat electrostatics with a 12 Å non-bonded cutoff. All bond lengths involving hydrogen atoms were constrained by using the SHAKE algorithm^60^ with a tolerance of 1.0 × 10^−5^ Å. The atomic coordinates were saved every 2 ps for further analysis. All of the trajectories were analyzed using the analysis module of AMBER 12.0 and some other developed specific MD analysis programs.

## Author Contributions

X.M.P. designed the experiments; L.Y.Z., X.C.X. and Y.Y. performed MD simulation and data analysis. L.Y.Z. wrote the main manuscript text and prepared all the figures. X.M.P., M.L.L. and Y.X.G. discussed the results and revised the manuscript. All authors contributed to discussions about the results and the manuscript.

## Supplementary Material

Supplementary Information Probing Immobilization Mechanism of alpha-chymotrypsin onto Carbon Nanotube in Organic Media by Molecular Dynamics Simulation

## Figures and Tables

**Table 1:** The component and standard errors of binding energy (in kcal mol^−1^) for α-ChT adsorbing on CNT in aqueous solution (WAT) and heptane (HEP) media

Contribution	WAT	HEP
Δ*E*_ele_^a^	0.00 ± 0.00	0.00 ± 0.00
Δ*E*_vdw_^b^	−122.07 ± 5.66	−67.54 ± 2.55
Δ*E*_int_^c^	0.00 ± 0.00	0.00 ± 0.00
Δ*E*_gas_^d^	−122.07 ± 5.66	−67.54 ± 2.55
Δ*G*_npsolv_^e^	−6.19 ± 0.36	−5.94 ± 0.19
Δ*G*_psolv_^f^	48.43 ± 2.53	25.96 ± 2.51
Δ*G*_solv_^g^	38.42 ± 2.29	20.02 ± 2.46
Δ*G*_ele_^h^	48.43 ± 2.53	25.96 ± 2.51
Δ*G*_binding_^i^	−83.65 ± 4.37	−47.53 ± 3.31

^a^Non-bonded electrostatic energy as calculated by the MM force field.

^b^Non-bonded van der walls contribution from MM force field.

^c^Internal energy arising from bond, angle, and dihedral terms in the MM force field.

^d^Total gas phase energy.

^e^Nonpolar contribution to the solvation free energy.

^f^Polar contribution to the solvation free energy calculated.

^g^Solvation free energy.

^h^Total electrostatic energy contribution to the binding energy.

^I^Binding energy.

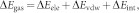

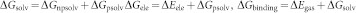

**Table 2:** Percentage occupation of hydrogen bonds (%) in aqueous solution and heptane media over the last 20 ns trajectories

System	cnt-wat	cnt-hep	free-wat	free-hep
NE2@His57**···**OG@Ser195	3.4%	99.7%	59.1%	83.1%
OD1@Asp102**···**ND1@His57	92.9%	93.8%	91.4%	98.8%
OD2@Asp102**···**ND1@His57	99.6%	99.9%	96.3%	99.8%

NE2@His57 denotes NE2 atom of His57 residue. OG@Ser195 denotes OG atom of Ser195 residue. OD2@Asp102 denotes OD2 atom of Asp102 residue. ND1@His57 denotes ND1 atom of His57 residue.
