# Supplementary material for: Probing Immobilization Mechanism of alpha-chymotrypsin onto Carbon Nanotube in Organic Media by Molecular Dynamics Simulation
Source: Sci Rep. 2015 Mar 19;5:9297. doi: 10.1038/srep09297 (PMC4365409; doi:10.1038/srep09297)
Supplement: Supplementary Information — Probing Immobilization Mechanism of alpha-chymotrypsin onto Carbon Nanotube in Organic Media by Molecular Dynamics Simulation [file srep09297-s1.doc]

Supplementary Information

Probing Immobilization Mechanism of alpha-chymotrypsin onto Carbon Nanotube in Organic Media by Molecular Dynamics Simulation

Liyun Zhang^a^, Xiuchan Xiao ^a^, Yuan Yuan^b^, Yanzhi Guo^a^, Menglong Li^a^, Xuemei Pu^a^,*

^a^Faculty of Chemistry, Sichuan University, Chengdu 610064, People’s Republic of China

^b^College of Management, Southwest University for Nationalities, Chengdu 610041, People’s Republic of China

1. **The similarity between three 200-ns parallel simulations with different starting velocities for every system**


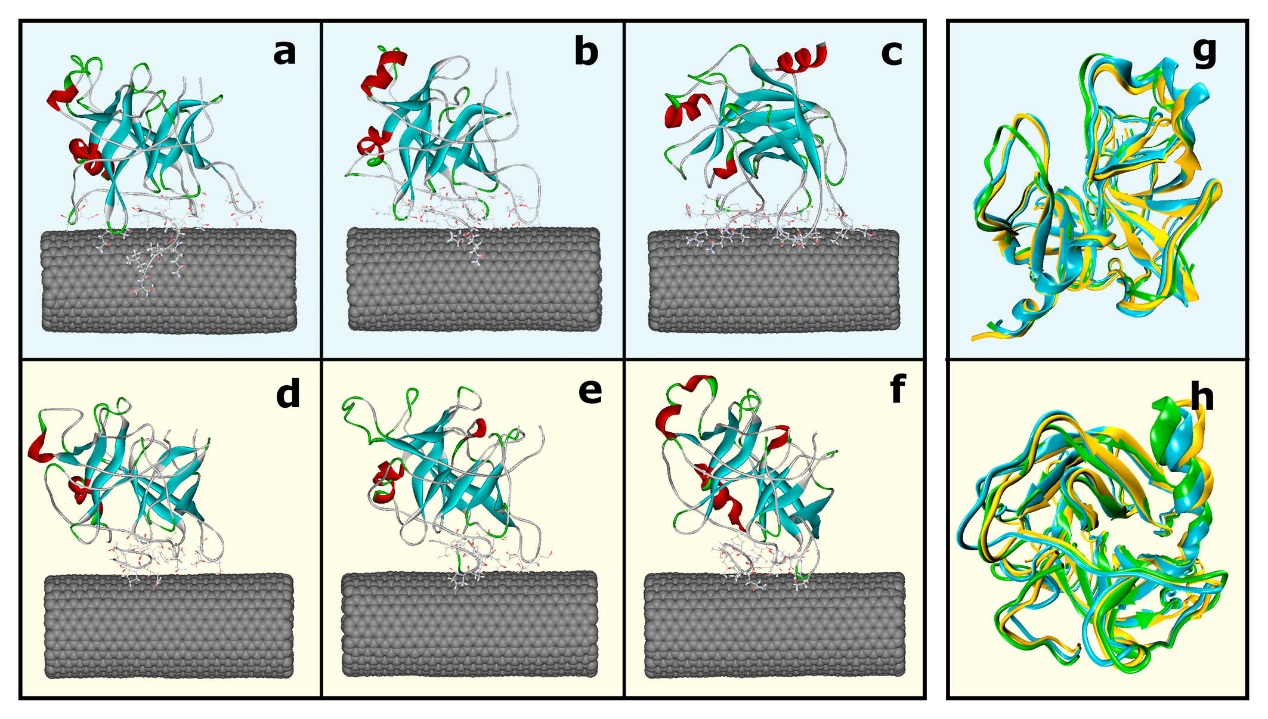


**Supplementary Figure S1** The representative conformations of the three parallel simulations for immobilized α-ChT in aqueous (a,b,c) and heptane(d,e,f) media. The superposition of the representative conformations of the free α-ChT derived from three parallel simulations in aqueous (g) and heptane (f) media. These representative conformations are derived from the frame with the lowest RMSD value from the corresponding average structure over the last 20 ns trajectories.

1. **The difference in the H-bonding of the active site of the enzyme bewteen the free enzyme and the immobilized one in heptane media**


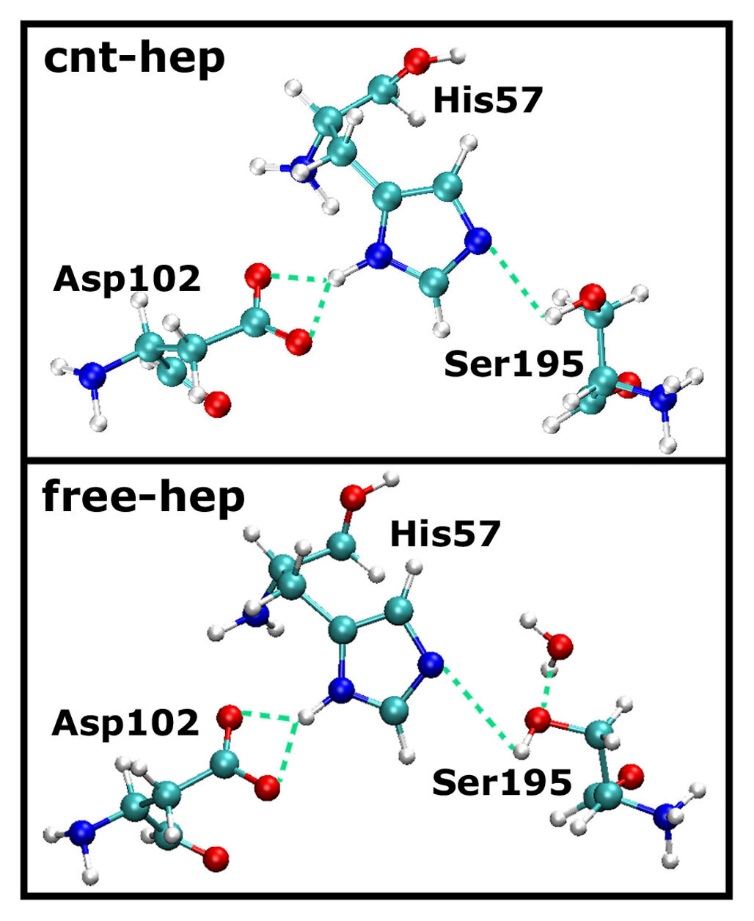


**Supplementary Figure S2** The H-bonding of the three catalytic residues of the free (free-hep) and immobilized (cnt-hep) enzymes in heptane media, derived from the last 20 ns trajectories. The hydrogen bonds are shown by green dotted line.

**3. Supplementary Table S1** Percentage occupation of the key hydrogen bonds (%) in S1 pocket in aqueous solution and heptane media over the last 20 ns trajectories.

| System | cnt-wat | cnt-hep | free-wat | free-hep |
| --- | --- | --- | --- | --- |
| OG@Ser190⋅⋅⋅OH@Tyr228  OG1@Thr140⋅⋅⋅ OH@Tyr228 | 69.3% | 43.8% | 71.8% |  |
|  |  | 84.5% |  |  |
| OG@Ser190⋅⋅⋅OG@Trp215 |  |  |  | 55.1% |
| O@H_2_O⋅⋅⋅OH@Tyr228 |  |  |  | 62.4% |
| OH@Tyr228⋅⋅⋅O@H_2_O |  |  |  | 36.8% |
| OG@Ser190 denotes OG atom of Ser190 residue. OH@Tyr228 denotes OH atom of Tyr228 residue. OG1@Thr140 denotes OG1 atom of Thr140 residue. OG@Trp215 denotes OG atom of Trp215 residue. O@H_2_O depicts O atom of the crystal water molecule. | | | | |
